# Supplementary material for: In situ infrared nanospectroscopy of the local processes at the Li/polymer electrolyte interface
Source: Nat Commun. 2022 Mar 17;13:1398. doi: 10.1038/s41467-022-29103-z (PMC8931078; doi:10.1038/s41467-022-29103-z)
Supplement: Supplementary file 1 — Supplementary Information [file 41467_2022_29103_MOESM1_ESM.pdf]

**SUPPLEMENTARY INFORMATION**

In situ Infrared Nanospectroscopy of Local Processes at the Li/Polymer Electrolyte Interface

Xin He[1,2,#], Jonathan M. Larson[1,#,*], Hans A. Bechtel[3*], Robert Kostecki[1*]

[1]Energy Storage and Distributed Resources Division, Lawrence Berkeley National Laboratory, Berkeley, CA 94720, USA

[2]Present address: School of Chemical Engineering, Sichuan University, 610017, Chengdu, PR China

[3]Advanced Light Source, Lawrence Berkeley National Laboratory, Berkeley, CA 94720, USA

[#]These authors contributed equally

[*]These authors jointly supervised this work. email: jmlarson@lbl.gov, HABechtel@lbl.gov, R_Kostecki@lbl.gov

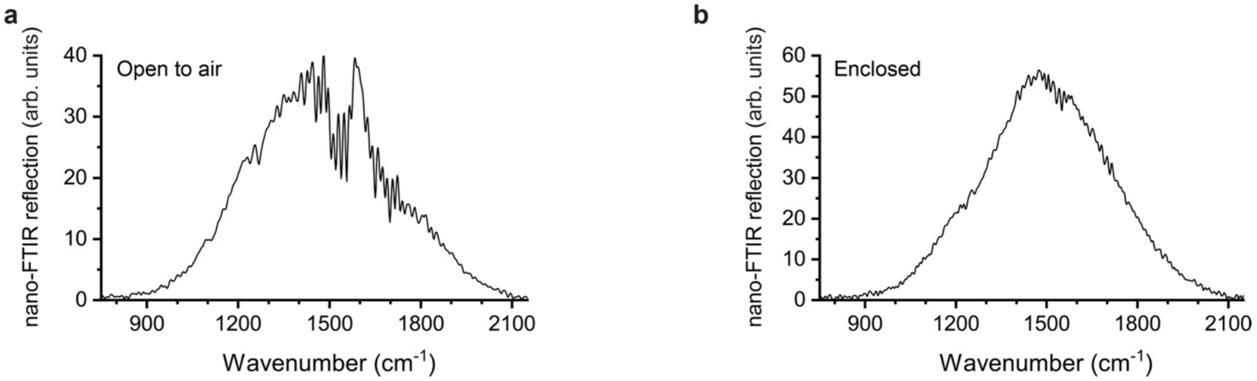


**Supplementary Fig. 1 Second harmonic of the near-field nano-FTIR amplitude signal from a silicon sample recorded: a** under ambient conditions and **b** in the environmental chamber filled with nitrogen.

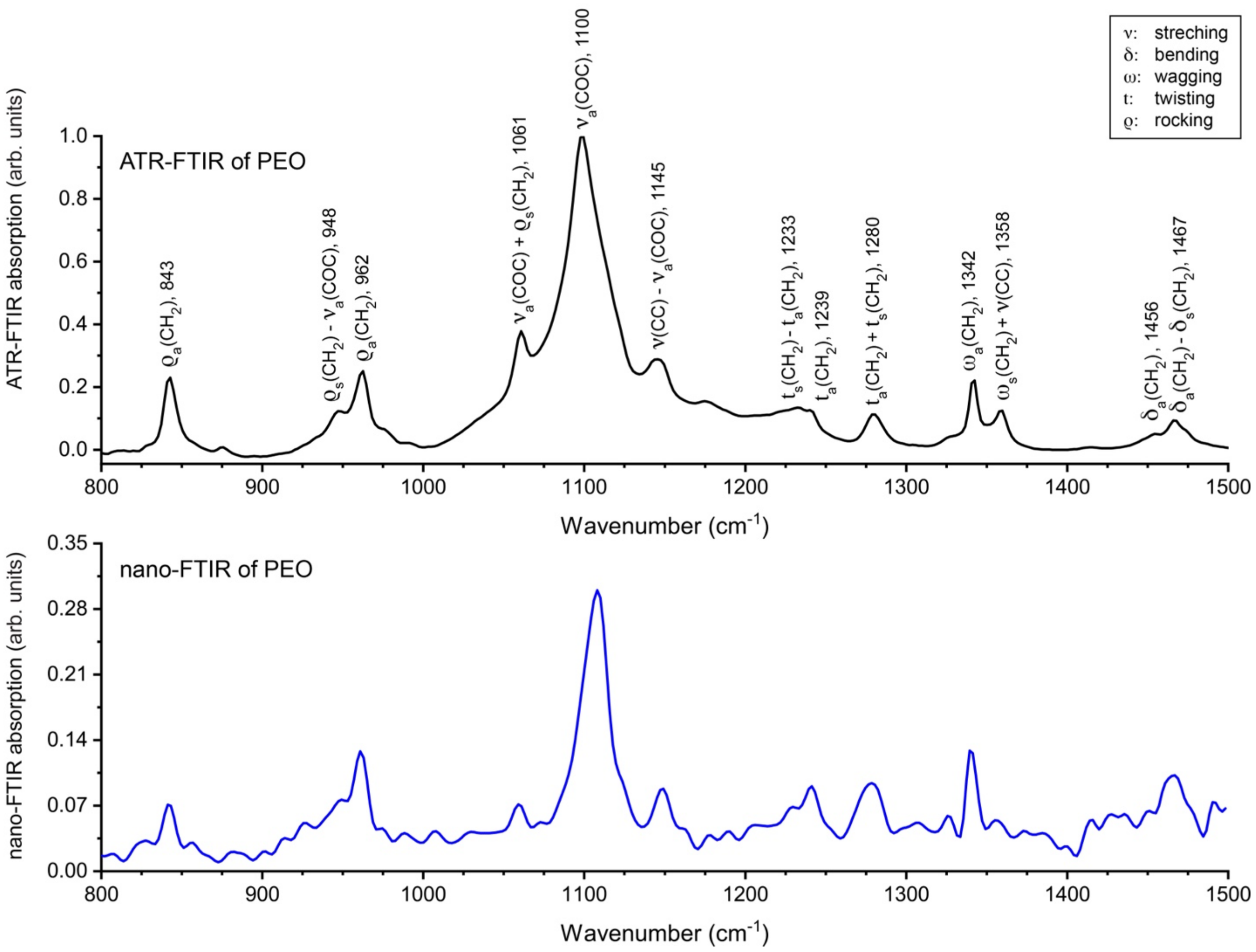


**Supplementary Fig. 2 ATR-FTIR (top) and nano-FTIR (bottom) of pure PEO**. Vibrational modes are assigned[1,2]. The ATR was collected in a custom glovebox. The nano-FTIR was collected at Beamline 2.4 of Berkeley Lab's Advanced Light Source.

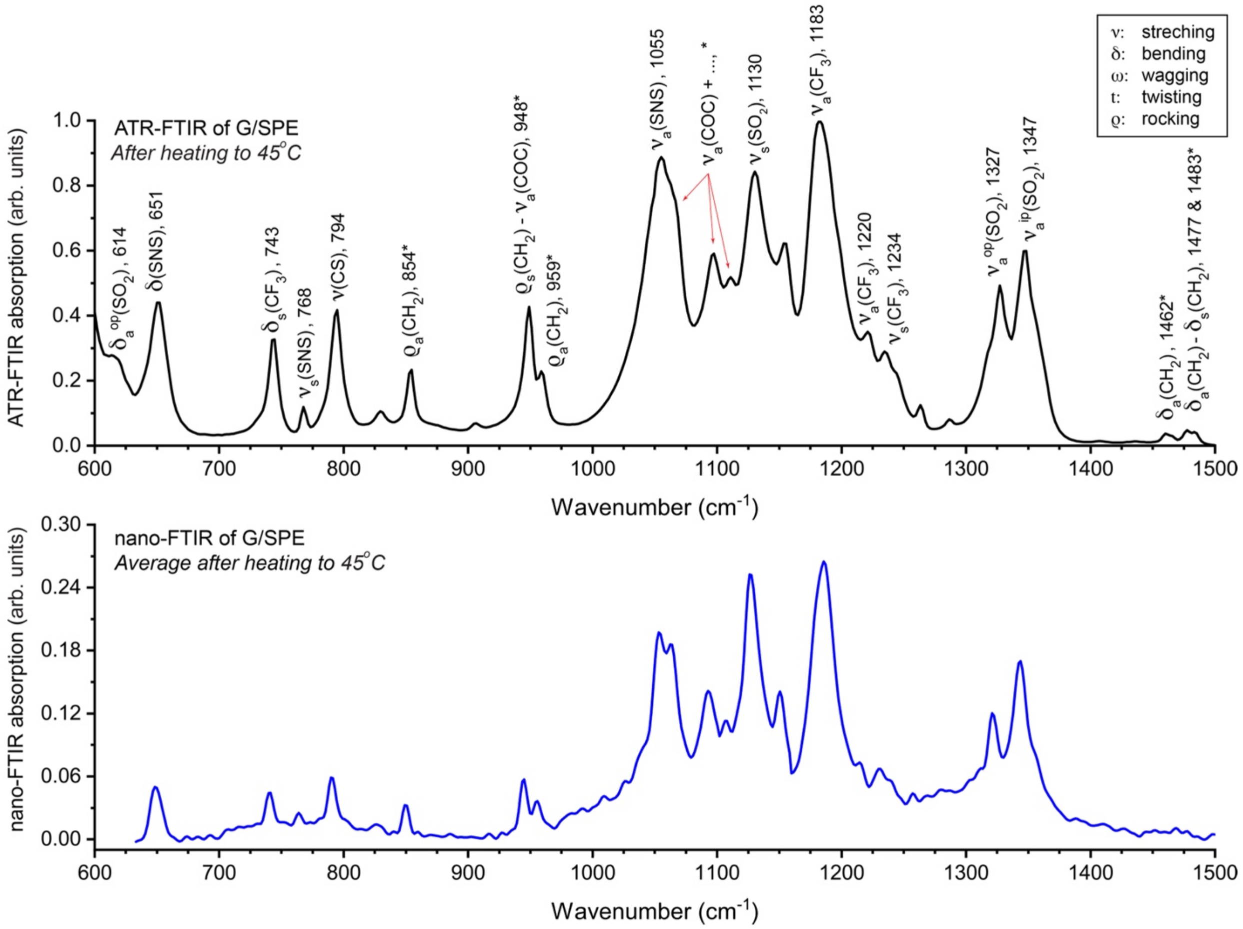


**Supplementary Fig. 3 ATR-FTIR (top) and nano-FTIR (bottom) of the heated interface.** Vibrational modes for the $PEO_{10}$:LiTFSI SPE are assigned[1-3]. The displayed nano-FTIR spectrum is the average of all nano-FTIR spectra collected in the heated case, while the ATR-FTIR is representative of both the pristine room temperature and after heating cases. The '*' symbol indicates modes attributable to PEO while a '+' sign indicates that additional modes are mixed with the primary one listed.

| Description of bands | PEO ($cm^{-1}$) | $TFSI^-$ ($cm^{-1}$) |
|---|---|---|
| out-of-phase asymmetric bending $SO_2$ | | 614 |
| bending SNS | | 651 |
| symmetric bending $CF_3$ | | 743 |
| symmetric stretching SNS | | 768 |
| symmetric stretching CS | | 794 |
| asymmetric rocking $CH_2$ | 854 | |
| symmetric rocking $CH_2$, mixed with aysmmetric stretching COC | 948 | |
| asymmetric rocking $CH_2$ | 959 | |
| asymmetric stretching SNS | | 1055 |
| asymmetric stretching COC | 1061, 1100, 1145 | |
| symmetric stretching $SO_2$ | | 1130 |
| asymmetric stretching $CF_3$ | | 1183 |
| asymmetric stretching $CF_3$ | | 1220 |
| symmetric stretching $CF_3$ | | 1234 |
| out-of-phase asymmetric stretching $SO_2$ | | 1327 |
| in-phase asymmetric stretching $SO_2$ | | 1347 |
| asymmetric bending $CH_2$ | 1462 | |
| asymmetric bending $CH_2$, mixed with symmetric bending $CH_2$ | 1477, 1483 | |

**Supplementary Table 1. Table of molecular vibrations in the SPE.** Vibrational modes for the $PEO_{10}$:LiTFSI SPE are assigned[1-3].

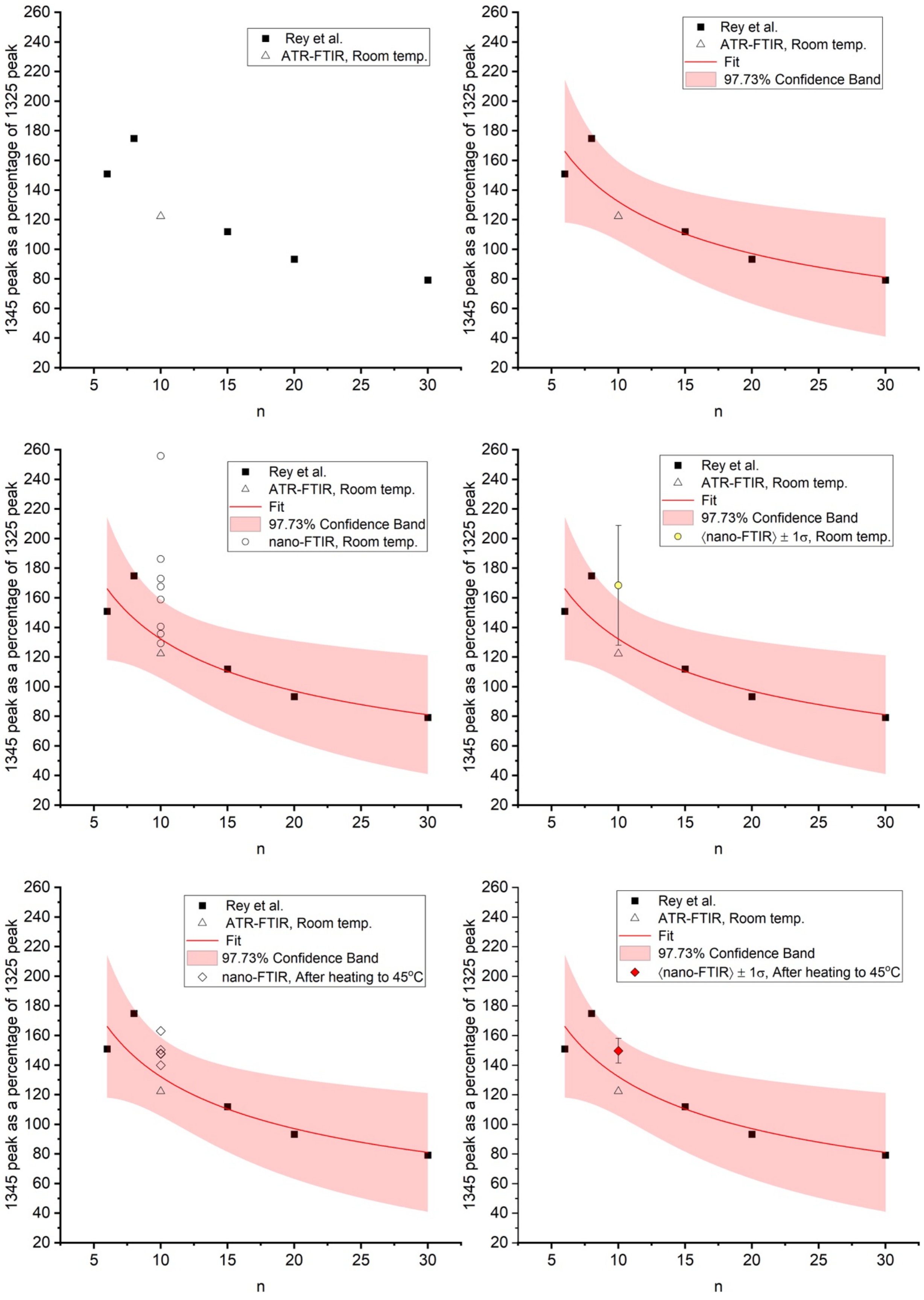

1345 peak as a percentage of 1325 peak
n
Rey et al.
ATR-FTIR, Room temp.
Fit
97.73% Confidence Band
nano-FTIR, Room temp.
⟨nano-FTIR⟩ ± 1σ, Room temp.
nano-FTIR, After heating to 45°C
⟨nano-FTIR⟩ ± 1σ, After heating to 45°C

**Supplementary Fig. 4 Analyses of the percent difference between absorption peaks of $\boldsymbol{\nu_a^{ip}(SO_2)}$ and $\boldsymbol{\nu_a^{op}(SO_2)}$ vibrational modes.** Top left is FTIR absorption data taken from both the literature[4] and our work. Top right is a simple power law fit to the data with a ±3 standard deviation confidence band overlaid. This fit serves as a characteristic tread to determine the relative concentration between the effective solvent and salt ($PEO_n$:LiTFSI) from IR absorption spectroscopy. The data, fit, and confidence band are plotted in all the rest of the panels. The middle left (bottom left) panel adds data taken from the spatially dependent nano-FTIR collected across the pristine room temperature (after heating to 45°C) interface. The middle right (bottom right) panel shows the average ratio of the nano-FTIR data in the pristine room temperature (after heating to 45°C) case with ±1 standard deviation error bars.

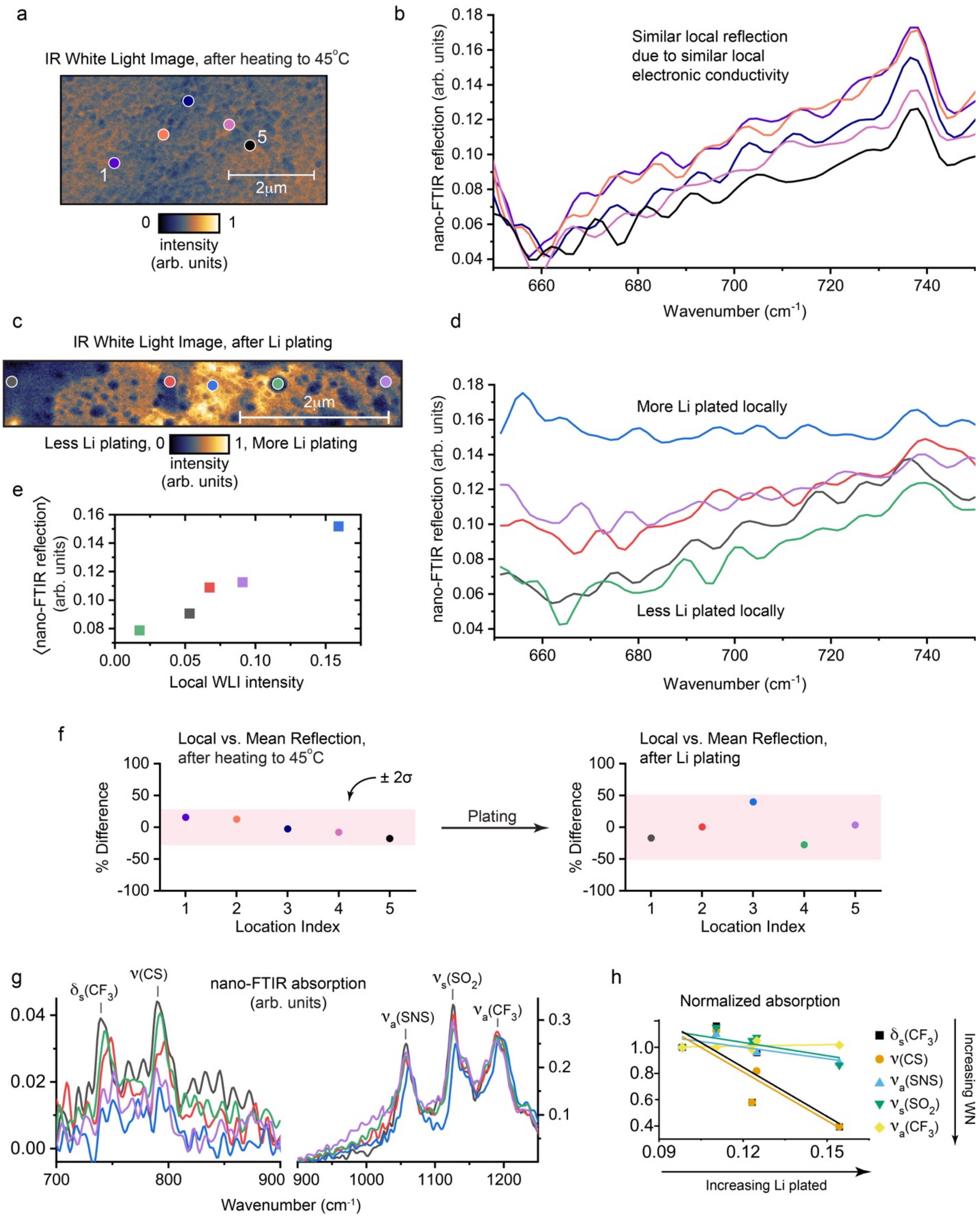


**Supplementary Fig. 5 Characterization of nanoscale heterogenous Li plating, and correlated screening, with both nano-FTIR reflection and absorption. a (c)** WL image of the heated (plated) interface. Colored circles indicate spatial locations of nano-FTIR acquisition. **b (d)** Local nano-FTIR reflection spectra of the heated (plated) interface. Spectra are color-matched with location markers on the

WL images. **e** Plot of the mean nano-FTIR reflection between 668 – 725 $cm^{-1}$ as a function of local IR WL image intensity. Square data points are color-matched with location markers on the WL images. **f** Plots of percent difference between locally averaged nano-FTIR reflection (between 668 – 725 $cm^{-1}$), and mean results from all spatial locations, in both the heated (left-hand side) and plated (right-hand side) states. Overlaid pink bars help to visualize ±2 standard deviations of the data sets. The standard deviation increases by 82% post plating, due to heterogenous Li plating. **g** Nano-FTIR absorption spectra of the interface after plating. Spectra are color-matched with location markers (**c**) and plotted in such a way to highlight the varied frequency-dependent screening effects observed after plating. **h** Normalized absorption as a function of local Li plating for various vibrational modes with different frequencies.

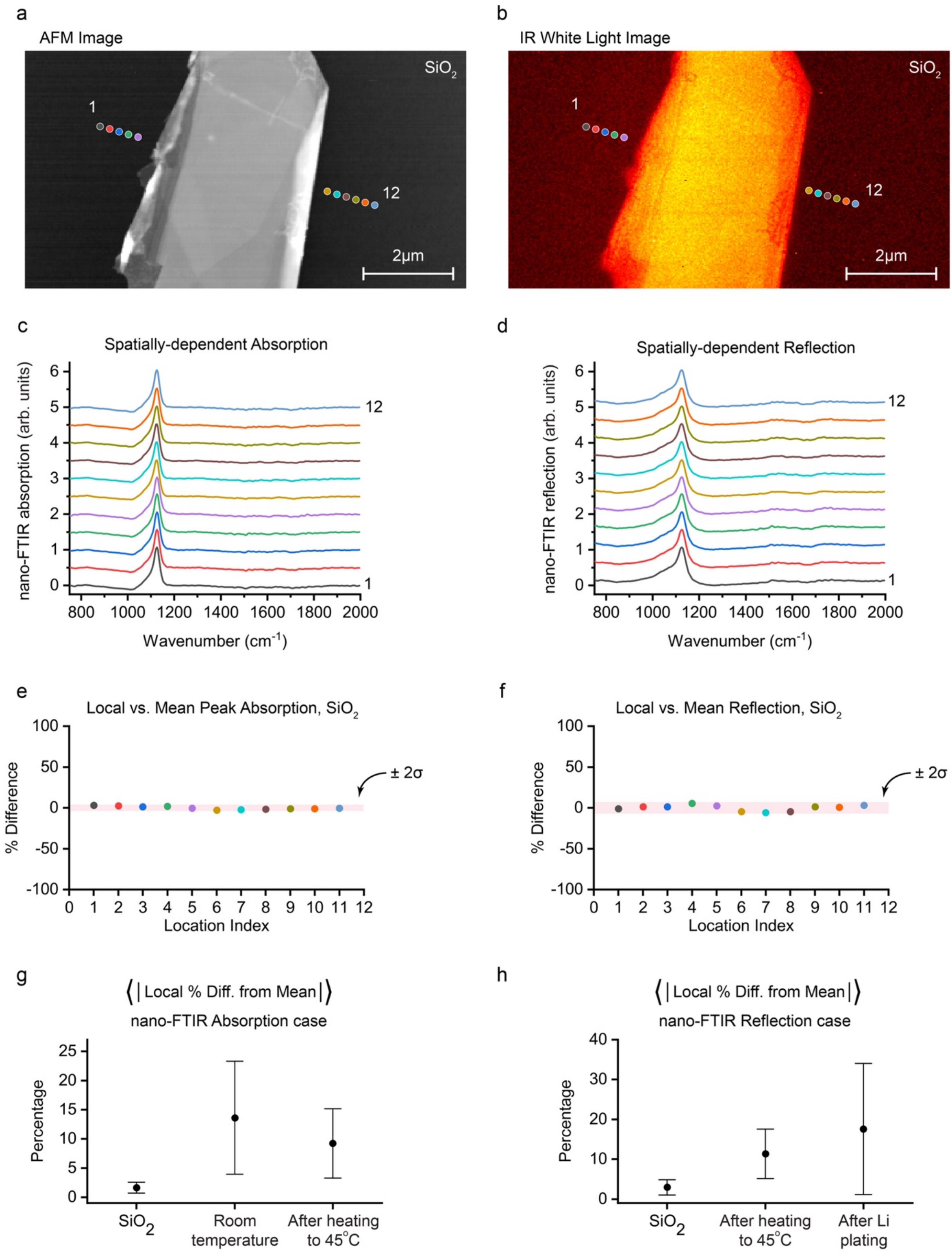


**Supplementary Fig. 6 Quantifying typical amounts of nano-FTIR absorption and reflection variation across a chemically homogeneous material ($SiO_2$) on a spatial scale of $10^2$ nm. a (b)** AFM (WL) image of graphene exfoliated on $SiO_2$. Colored circles indicate spatial locations of nano-FTIR acquisition. **c (d)**

Nano-FTIR absorption (reflection) across $SiO_2$. Colors and numbers of spectra match the corresponding numbers and colors specifying location in the AFM and WL images. Spectra were collected at Berkeley Lab's Advanced Light Source on Beamline 2.4. **e** Scatterplot of $SiO_2$ phonon absorption peak value as a percentage of mean peak value, plotted as a function of location (~$10^2$ nm separations). Overlaid pink bar visualizes ±2 standard deviations of the entire data set. **f** Plots of percent difference between locally averaged nano-FTIR reflection (between 1850 – 2000 $cm^{-1}$), and mean results from all spatial locations. The overlaid pink bar is ±2 standard deviations of the data set. **g (h)** Scatterplot of the averaged absolute value percent differences from mean absorption (reflection) in the case of $SiO_2$, pristine room temperature state of the SSPB device, and after heating state of the SSPB device ($SiO_2$, heated state of the SSPB device, and plated state of the SSPB device).

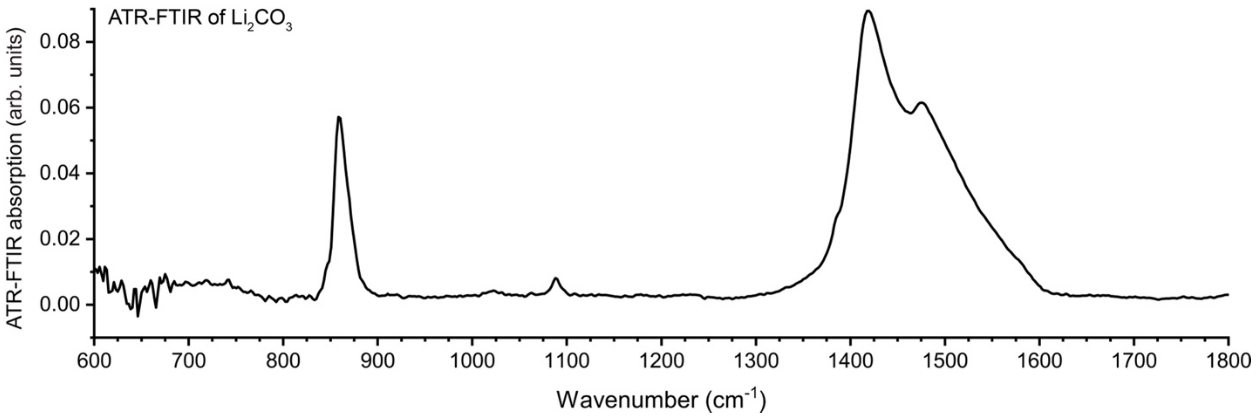


**Supplementary Fig. 7 ATR-FTIR of lithium carbonate.**

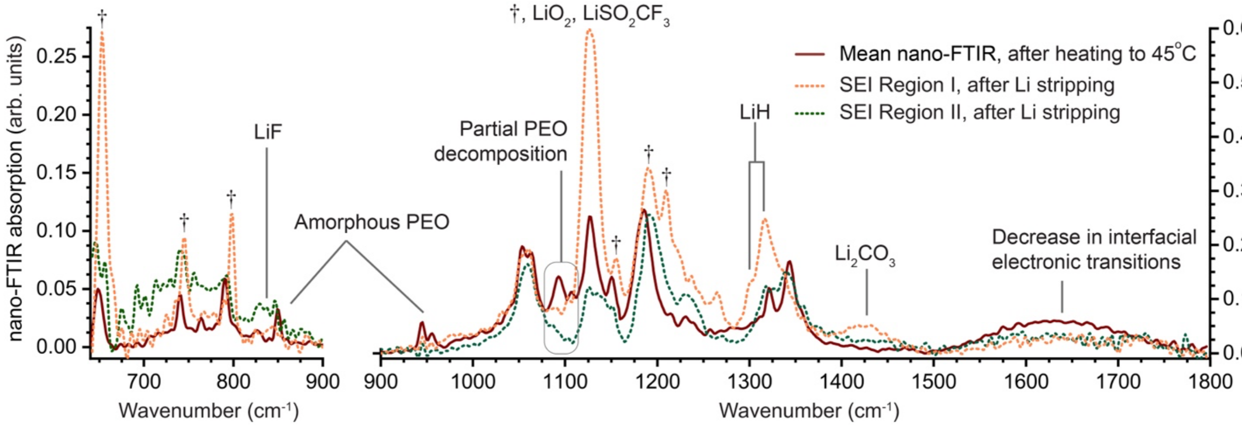


**Supplementary Fig. 8 Nano-FTIR after heating and after Li stripping.** Average nano-FTIR spectra of the interface after heating to 45°C and after Li stripping from the two SEI regions indicated by the colored locations in Fig. 4a. Presented data is the same data from Fig. 4b, only plotted on a larger scale, and with

various findings mentioned in the main text highlighted. Daggers mark absorption bands of $TFSI^-$ which have been enhanced after platting, likely indicating SEI Region I was enriched with $TFSI^-$ by anion migration toward the interface during Li stripping.

## Supplementary References


1. Yoshihara, T., Tadokoro, H. & Murahashi, S. Normal vibrations of the polymer molecules of helical conformation. IV. polyethylene oxide and polyethylene‐d4Oxide. *J. Chem. Phys.* **41**, 2902-2911 (1964).
2. Li, X. & Hsu, S. L. An analysis of the crystallization behavior of poly(ethylene oxide) poly(methyl methacrylate) blends by spectroscopic and calorimetric techniques. *J. Polym. Sci. Polym. Phys. Ed.* **22**, 1331-1342 (1984).
3. Rey, I. *et al.* Spectroscopic and theoretical study of (CF3SO2)2N- (TFSI-) and (CF3SO2)2NH (HTFSI). *J. Phys. Chem. A* **102**, 3249-3258 (1998).
4. Rey, I., Lassegues, J. C., Grondin, J. & Servant, L. Infrared and raman study of the PEO-LiTFSI polymer electrolyte. *Electrochim. Acta* **43**, 1505-1510 (1998).
